# Supplementary material for: PIF transcription factors link a neighbor threat cue to accelerated reproduction in Arabidopsis
Source: Nat Commun. 2019 Sep 5;10:4005. doi: 10.1038/s41467-019-11882-7 (PMC6728355; doi:10.1038/s41467-019-11882-7)
Supplement: Supplementary file 1 — Supplementary Information [file 41467_2019_11882_MOESM1_ESM.pdf]

a

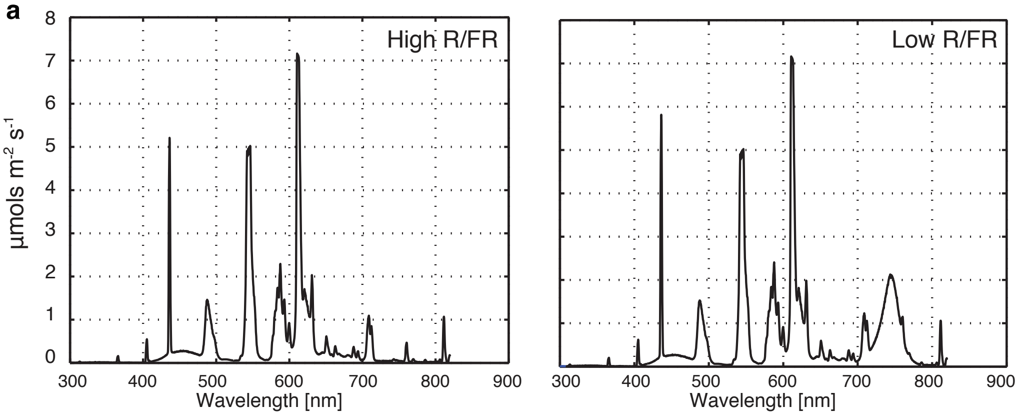


b

| Condition | PAR | Blue | Red | Far-Red | R/FR ratio |
|---|---|---|---|---|---|
| High R/FR | 212 | 69.1 | 13.2 | 10.7 | 1.231 |
| Low R/FR | 215 | 69.0 | 13.3 | 65.9 | 0.201 |

**Supplementary Figure 1**

**a** Light spectra and fluence rate of high R/FR ratio and low R/FR ratio light conditions obtained with OceanOptics USB2000+ spectrometer in CLF plant growth incubator model AR-41L equipped with fluorescent light bulbs and supplemental far-red light-emitting diodes (LED). **b** Light intensities (µmoles $m^{-2}$ $s^{-1}$) of photosynthetically active radiation (PAR, between 400-700 nm), blue (400-500 nm), red (640-700 nm), far-red (700-760 nm) are listed on the table.

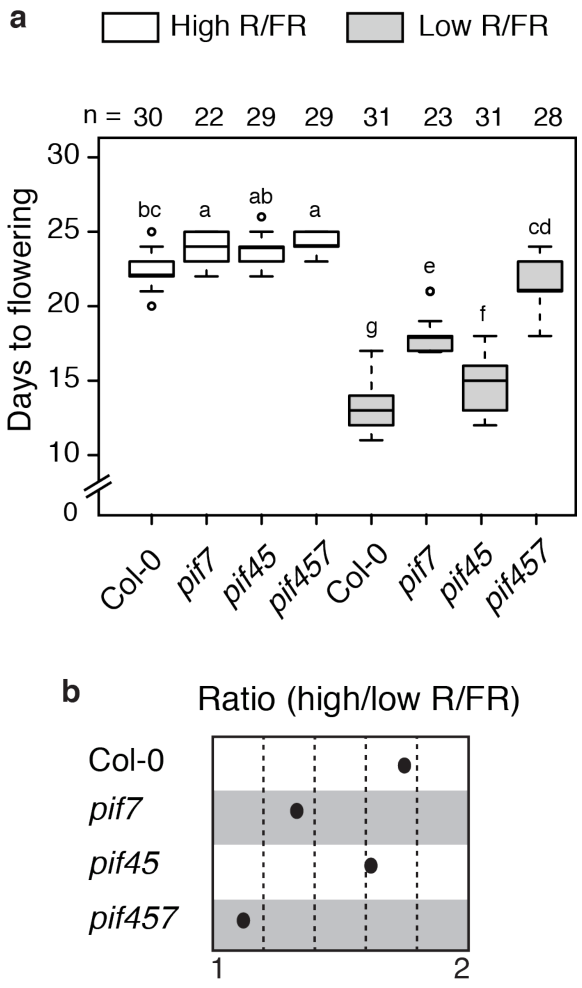


**Supplementary Figure 2**

PIFs mediate flowering in low R/FR. Flowering time represented as number of days to flowering after sowing of plants growing under LD at 22°C in high and low R/FR **a** and the ratio of days to flowering in high *vs* low R/FR **b**. Plants were grown for 5 days high R/FR for complete de-etiolation and either kept in high R/FR or shifted to low R/FR on day 6 until the onset of flowering. Letters represent the significance groups at p-value < 0.01 using ANOVA followed by Tukey honestly significant difference (HSD) test. n represent the number of plants phenotyped. Boxplots were created using the online BoxPlotR [1]; center line, median; box limits, upper and lower quartiles; whiskers, 1.5x interquartile range (IQR); dots, outliers. n represents the number of plants phenotyped.

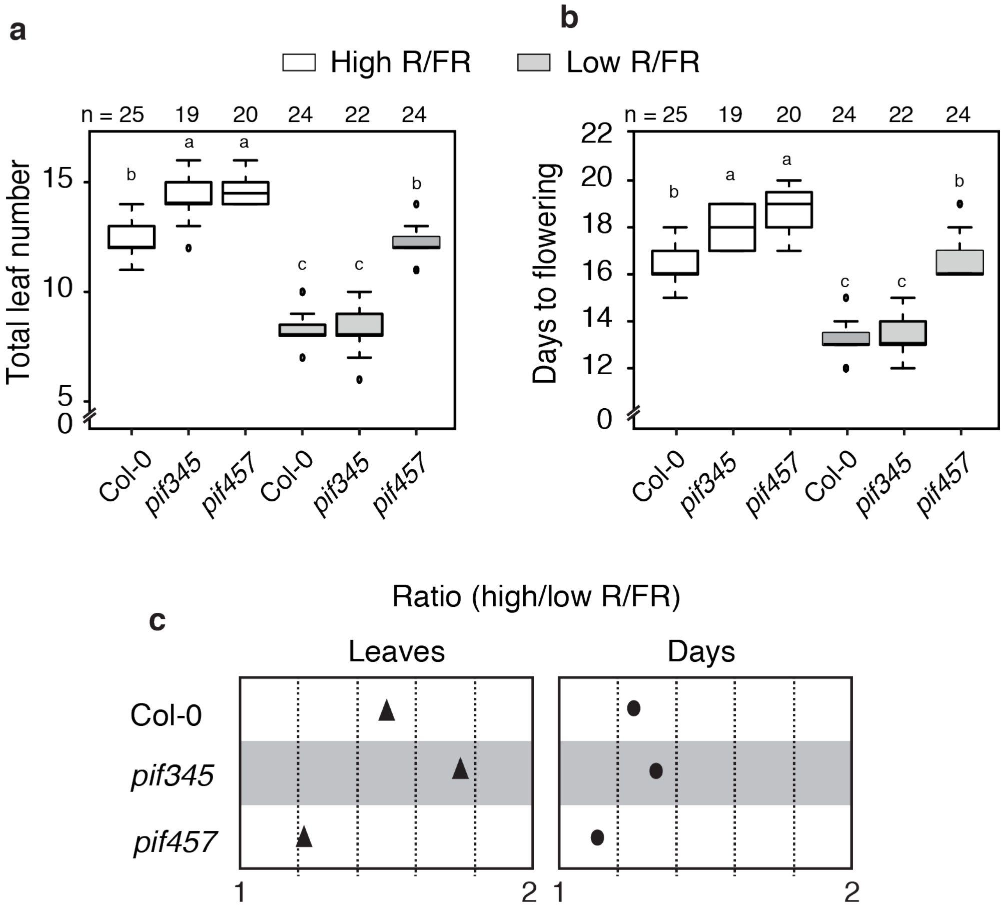


**Supplementary Figure 3**
Flowering time phenotype represented as total leaf number **a** and days to flowering **b** after bolting. Plants were grown under LD at 22°C in high R/FR for 5 days and either kept in the same condition or shifted to low R/FR on day 6 until the onset of flowering. **c** Ratio of total leaf number and days to flowering in high *vs* low R/FR represented in **a** and **b**. Letters represent the significance groups at p-value < 0.01 using ANOVA followed by Tukey honestly significant difference (HSD) test. n represent the number of plants phenotyped. Boxplots were created using the online BoxPlotR [1]; center line, median; box limits, upper and lower quartiles; whiskers, 1.5x interquartile range (IQR); dots, outliers.

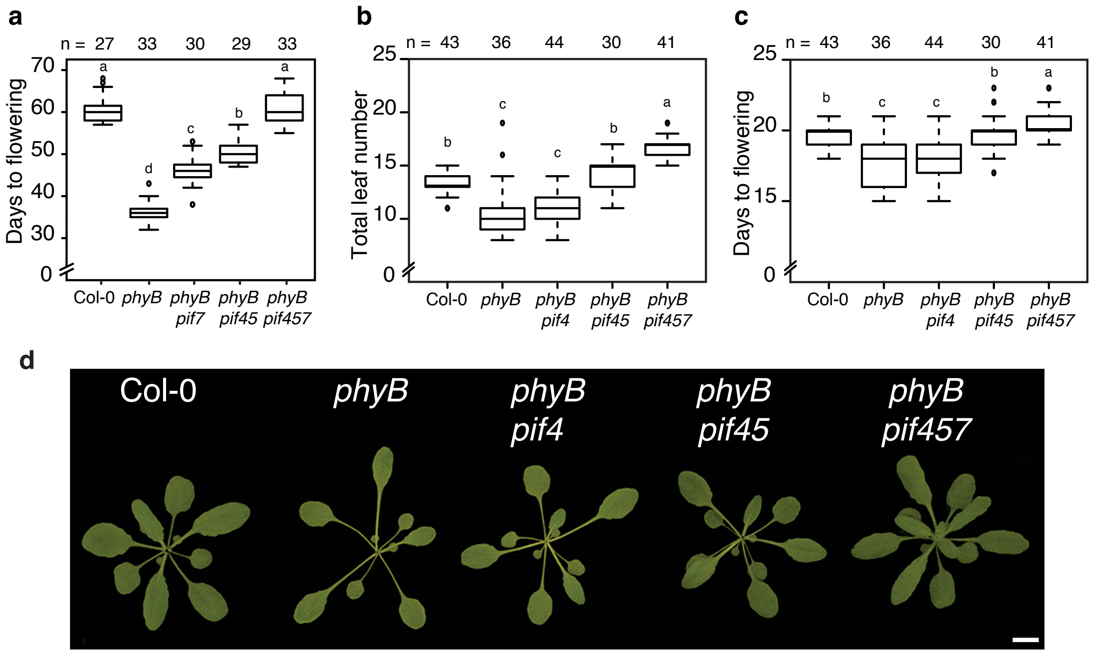


**Supplementary Figure 4**

**a** Days to flowering after sowing of Col-0, *phyB*, *phyB pif7*, *phyB pif4 pif5* and *phyB pif4 pif5 pif7* growing under SD at 22°C in high R/FR. Total leaf number **b** and days to flowering after sowing **c** of Col-0, *phyB*, *phyB pif4*, *phyB pif4 pif5* and *phyB pif4 pif5 pif7* mutants growing under LD at 22°C in high R/FR. **d** 25-day-old representative plants grown under LD. Scale bar correspond to 1 cm. Letters represent the significance groups at p-value < 0.01 using ANOVA followed by Tukey honestly significant difference (HSD) test. n represent the number of plants phenotyped. Boxplots were created using the online BoxPlotR [1]; center line, median; box limits, upper and lower quartiles; whiskers, 1.5x interquartile range (IQR); dots, outliers.

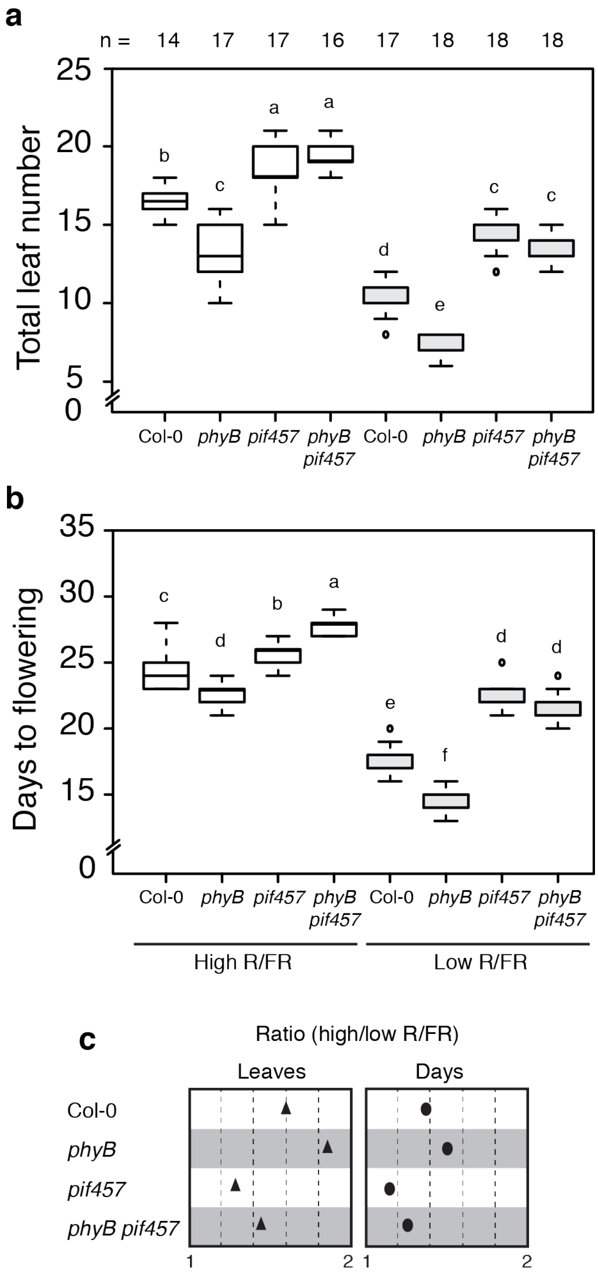


**Supplementary Figure 5**
PIF4, PIF5 and PIF7 mediate flowering transition downstream of phyB in high and low R/FR. The flowering time phenotype is represented as total leaf number **a** and days to flowering **b** after bolting. Plants were grown under LD at 22°C in high R/FR for 5 days and either kept in the same condition or shifted to low R/FR until the onset of flowering. **c** Ratio of total leaf number and days to flowering in high *vs* low R/FR represented in **a** and **b**. Letters represent the significance groups at p-value < 0.01 using ANOVA followed by Tukey honestly significant difference (HSD) test. n represent the number of plants phenotyped. Boxplots were created using the online BoxPlotR [1]; center line, median; box limits, upper and lower quartiles; whiskers, 1.5x interquartile range (IQR); dots, outliers.

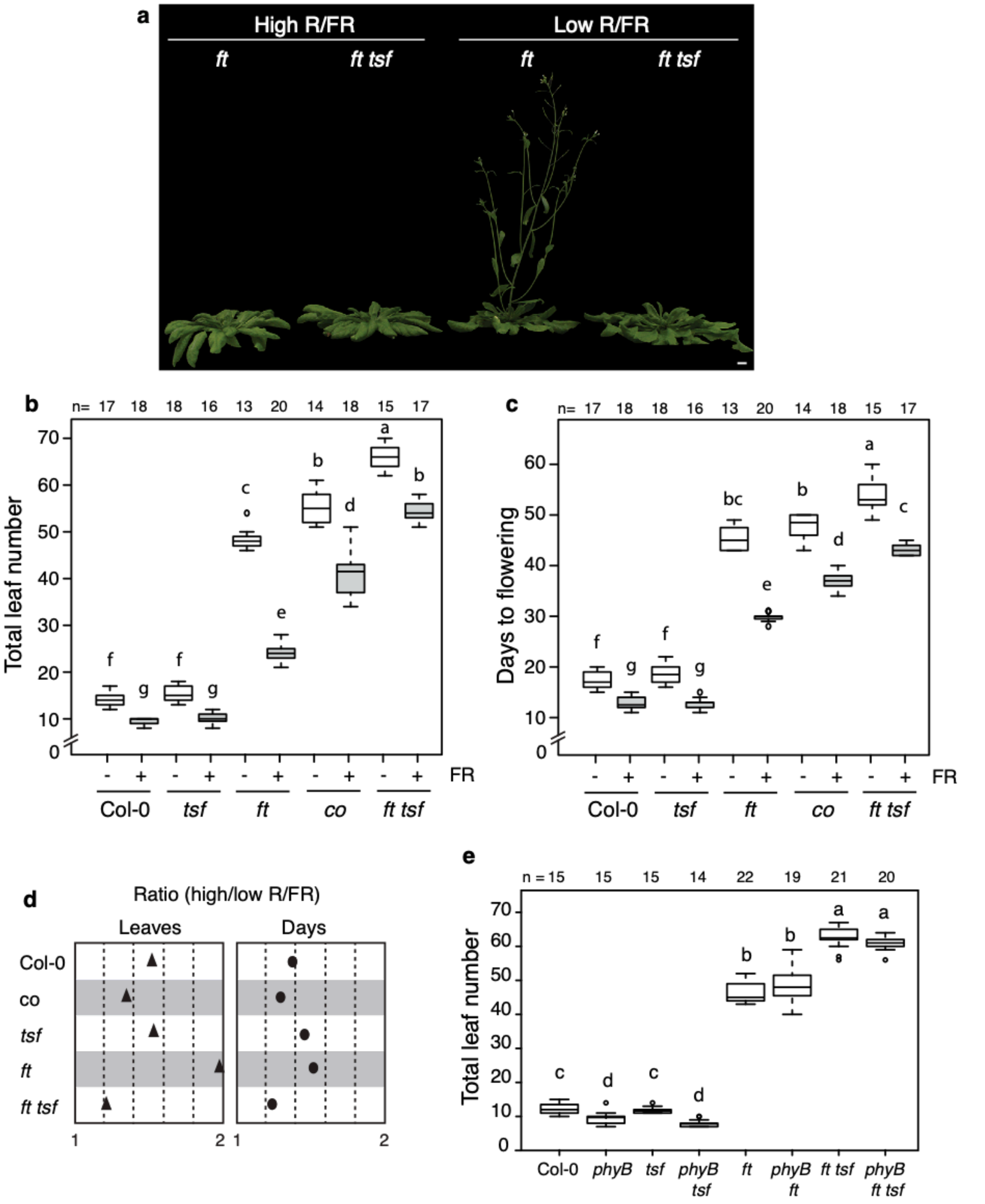


**Supplementary Figure 6**

**a** Representative plants of *ft* single and *ft tsf* double mutant genotypes grown under LD at 22°C in high R/FR and low R/FR. Plants were grown under LD at 22°C in high R/FR for 5 days and either kept in the same condition or shifted to low R/FR until the onset of flowering. Flowering time phenotype was represented as total leaf number **b** and days to flowering after sowing **c**. **d** Ratio of total leaf number and days to flowering in high *vs* low R/FR represented in **b** and **c**. **e** Flowering time phenotype of Col-0, *phyB*, *tsf*, *phyB tsf*, *ft*, *phyB ft*, *ft tsf* and *phyB ft tsf* under LD at 22°C in high R/FR was represented as total leaf number after bolting. FR, supplemental far-red light. Letters represent the significance groups at p-value < 0.01 using ANOVA followed by Tukey honestly significant difference (HSD) test. n represent the number of plants phenotyped. Boxplots were created using the online BoxPlotR [1]; center line, median; box limits, upper and lower quartiles; whiskers, 1.5x interquartile range (IQR); dots, outliers. Scale bar correspond to 1 cm.

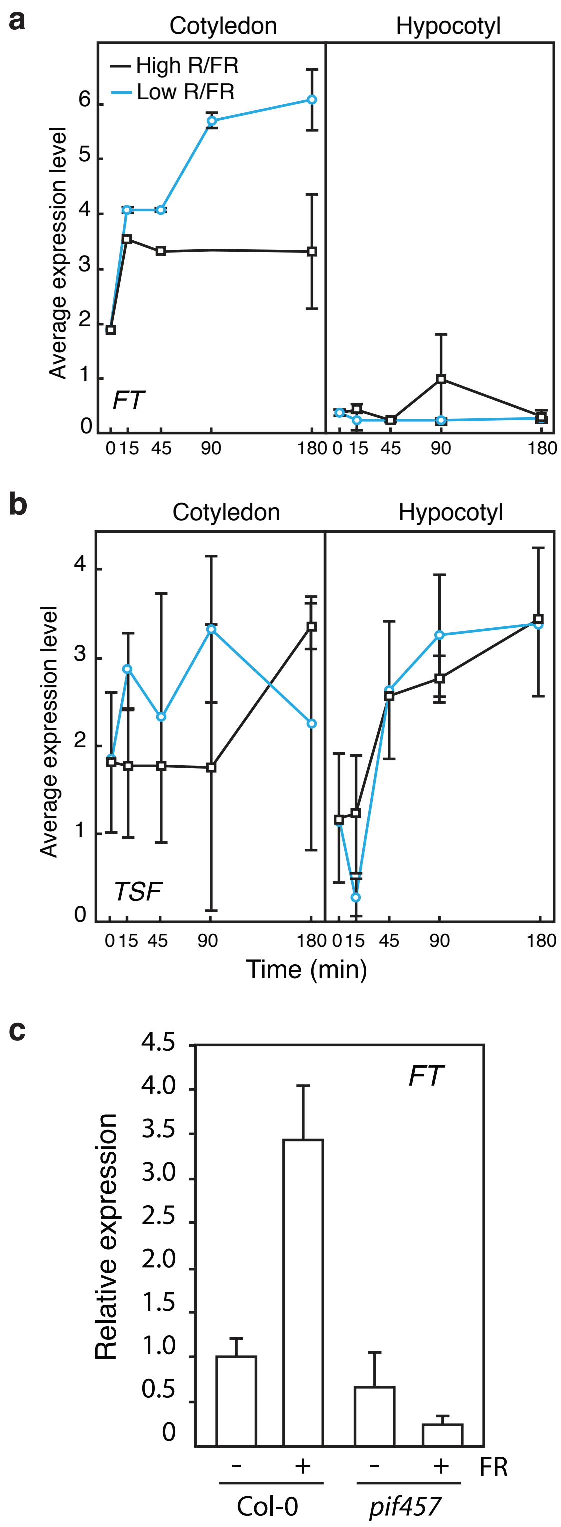


**Supplementary Figure 7**

Short-term *FT* up-regulation in low R/FR. *FT* **a** and *TSF* **b** RNA-seq data of relative expression in hypocotyl and cotyledon samples before (0) and 15, 45, 90 and 180 minutes after shift from high to low R/FR. RNA-seq expression data was obtained from [2]. **c** *FT* transcript level using quantitative real-time PCR (RT-qPCR) of Col-0 and *pif4 pif5 pif7* plants growing under LD at 22°C. Plants were grown for 5 days in high R/FR and on day 6 either kept in standard high R/FR or shifted to low R/FR at ZT2. Samples were harvested 180 minutes after shift to low R/FR (ZT 5). FR, supplemental far-red light. Error bars represent the standard deviation of three biological and three technical replicates.

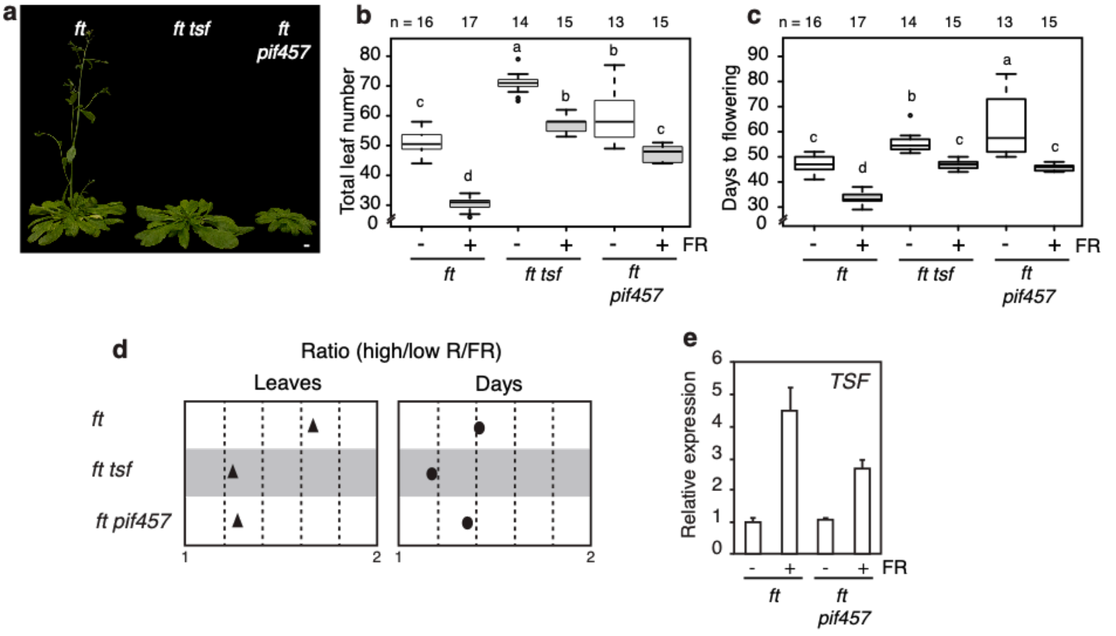


**Supplementary Figure 8**
**a** Representative 50-day-old *ft*, *ft tsf* and *ft pif4 pif5 pif7* plants grown under LD at 22°C in low R/FR. Plants were grown under LD at 22°C in high R/FR for 5 days and either kept in the same condition or shifted to low R/FR on day 6 until the onset of flowering. Flowering time was represented as total leaf number **b** and days to flowering after sowing **c** in high and low R/FR. FR, supplemental far-red light. **d** Ratio of total leaf number and days to flowering after sowing in high *vs* low R/FR. **e** *TSF* mRNA levels in *ft* and *ft pif4 pif5 pif7* 19 days after sowing. Plants were grown under LD at 22°C in high R/FR for 5 days and either kept in the same condition or shifted to low R/FR on day 6. Letters represent the significance groups at p-value < 0.01 using ANOVA followed by Tukey honestly significant difference (HSD) test. n represent the number of plants phenotyped. Boxplots were created using the online BoxPlotR [1]; center line, median; box limits, upper and lower quartiles; whiskers, 1.5x interquartile range (IQR); dots, outliers. Scale bar correspond to 1 cm.

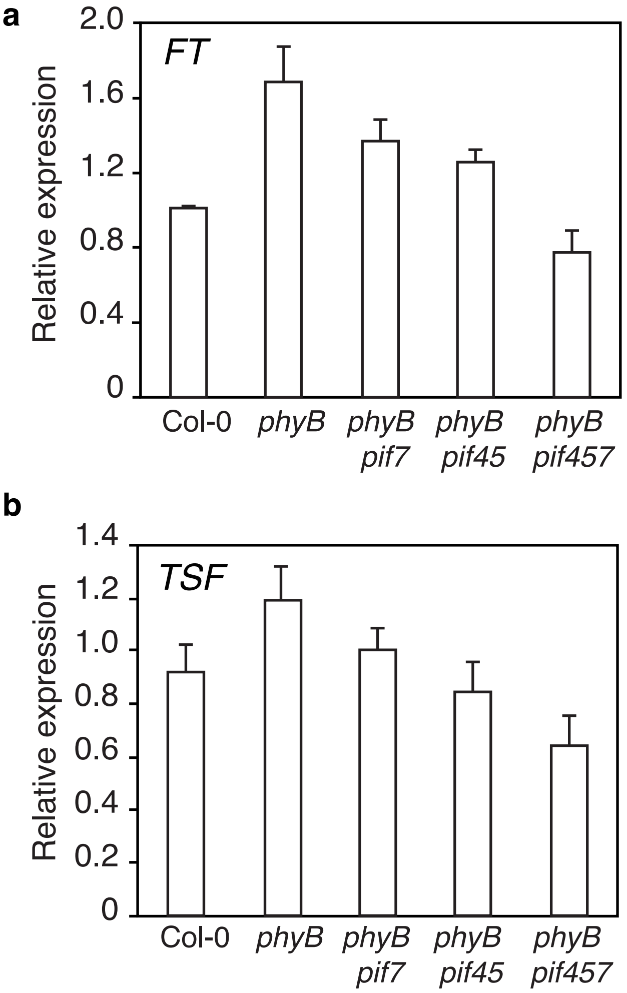


**Supplementary Figure 9**

*FT* **a** and *TSF* **b** mRNA quantification using quantitative real-time PCR (RT-qPCR) of Col-0, *phyB*, *phyB pif7*, *phyB pif4 pif5* and *phyB pif4 pif5 pif7* plants growing under LD at 22°C. Plants were grown high R/FR at 22°C and samples were harvested 10 days after sowing at ZT 15-16. Error bars represent the standard deviation of three biological and three technical replicates.

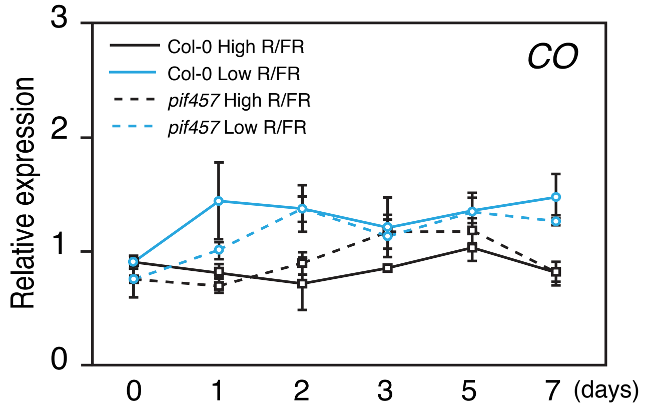


**Supplementary Figure 10**

*CO* mRNA level using quantitative real-time PCR (RT-qPCR) after shift from high to low R/FR in Col-0 and *pif4 pif5 pif7*. Plants were grown on soil for 5 days under LD at 22°C in high R/FR and samples were harvested at ZT 15-16 before (0) and 1, 2, 3, 5, and 7 days after shift to low R/FR. Error bars represent 2x SEM.

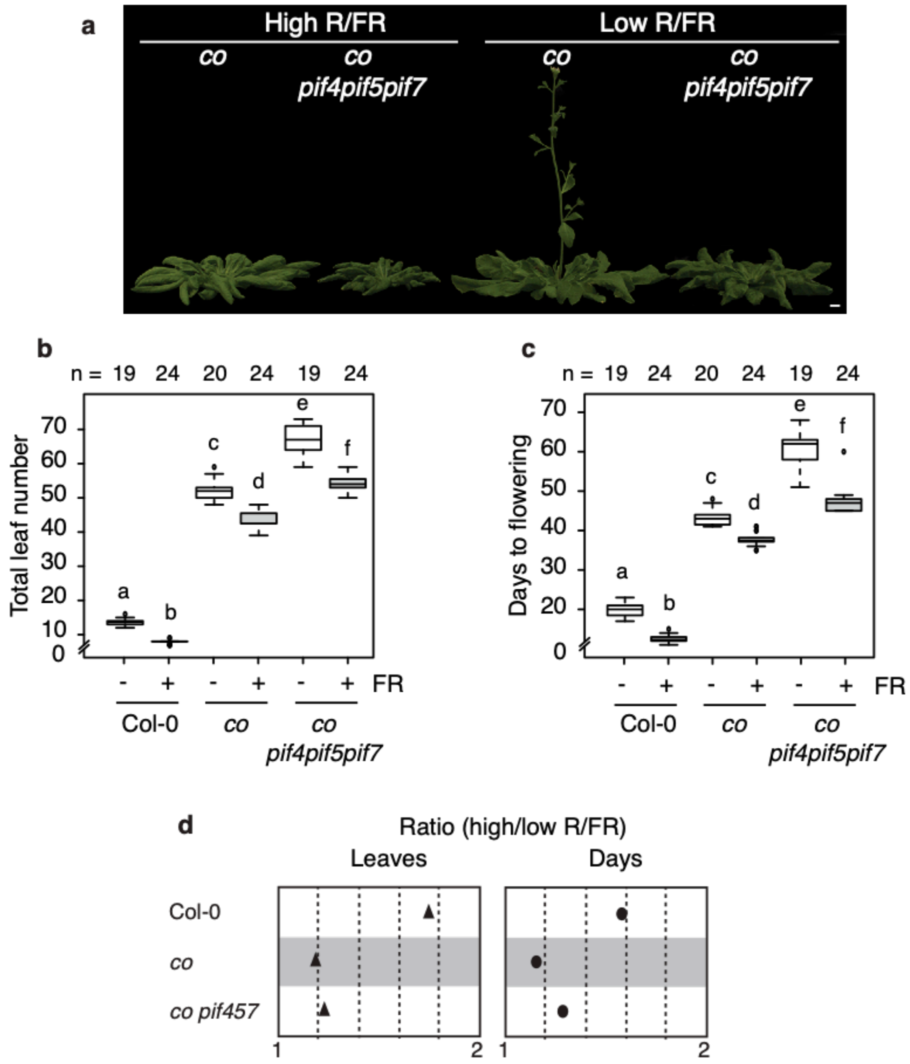


**Supplementary Figure 11**

**a** Representative plants of *co* single and *co pif4 pif5 pif7* quadruple mutant were grown under LD at 22°C in high R/FR and low R/FR. Plants were grown under LD at 22°C in high R/FR for 5 days and either kept in the same condition or shifted to low R/FR on day 6 until the onset of flowering. Flowering time phenotype was represented as total leaf number **b** and days to flowering after bolting **c**. **d** Ratio of total leaf number and days to flowering of plants growing under high *vs* low R/FR represented in **b** and **c**. FR, supplemental far-red light. Letters represent the significance groups at p-value < 0.01 using ANOVA followed by Tukey honestly significant difference (HSD) test. n represent the number of plants phenotyped. Boxplots were created using the online BoxPlotR [1]; center line, median; box limits, upper and lower quartiles; whiskers, 1.5x interquartile range (IQR); dots, outliers. Scale bar correspond to 1 cm.

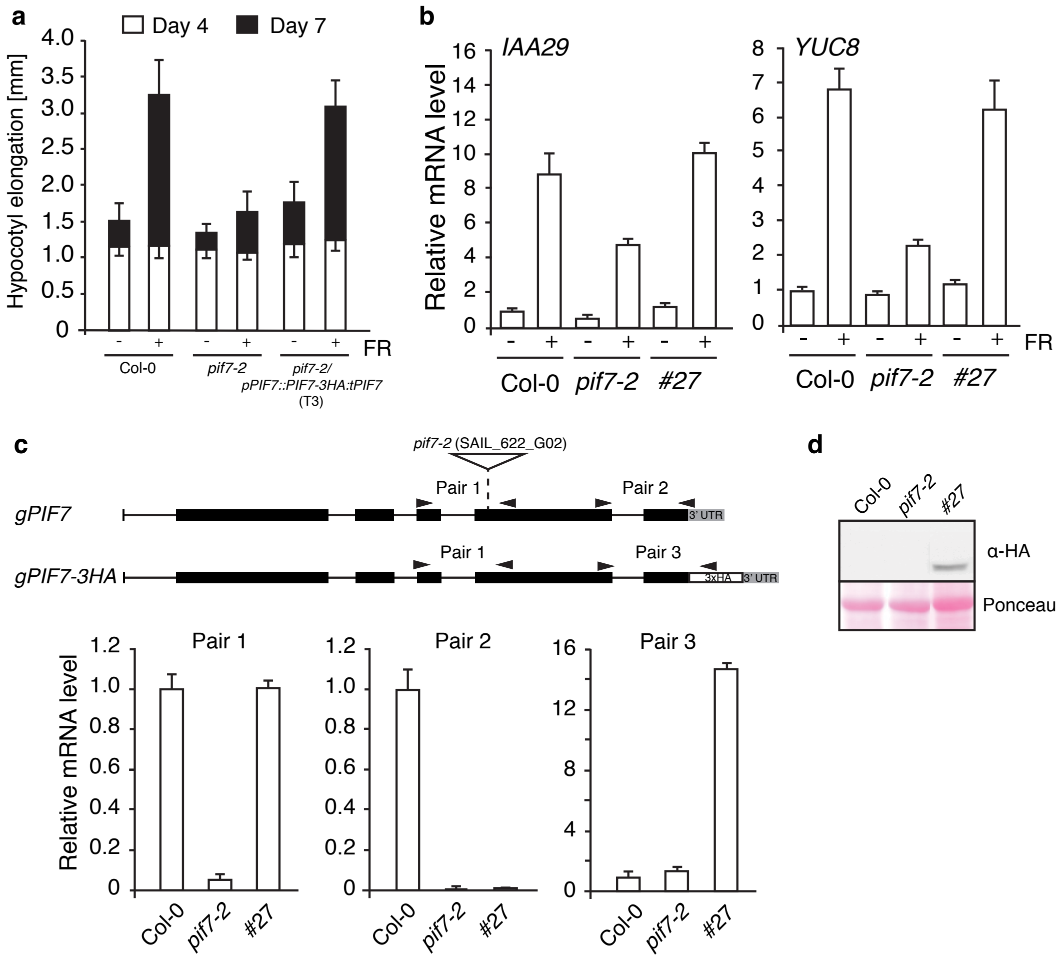


## Supplementary Figure 12

*pPIF7::PIF7-3HA-tPIF7* (*gPIF7*, labelled #27 on the figure) recues the *pif7-2* short hypocotyl phenotype **a** and the expression of marker genes *IAA29* and *YUC8* **b** in low R/FR. Transgene *gPIF7* expression was confirmed both using quantitative real time PCR (RT-qPCR) with both gene- and transgene-specific oligonucleotides **c** and western blot probed against α-HA **d**. Error bars represent standard deviation of three biological replicates. FR, supplemental far-red light.

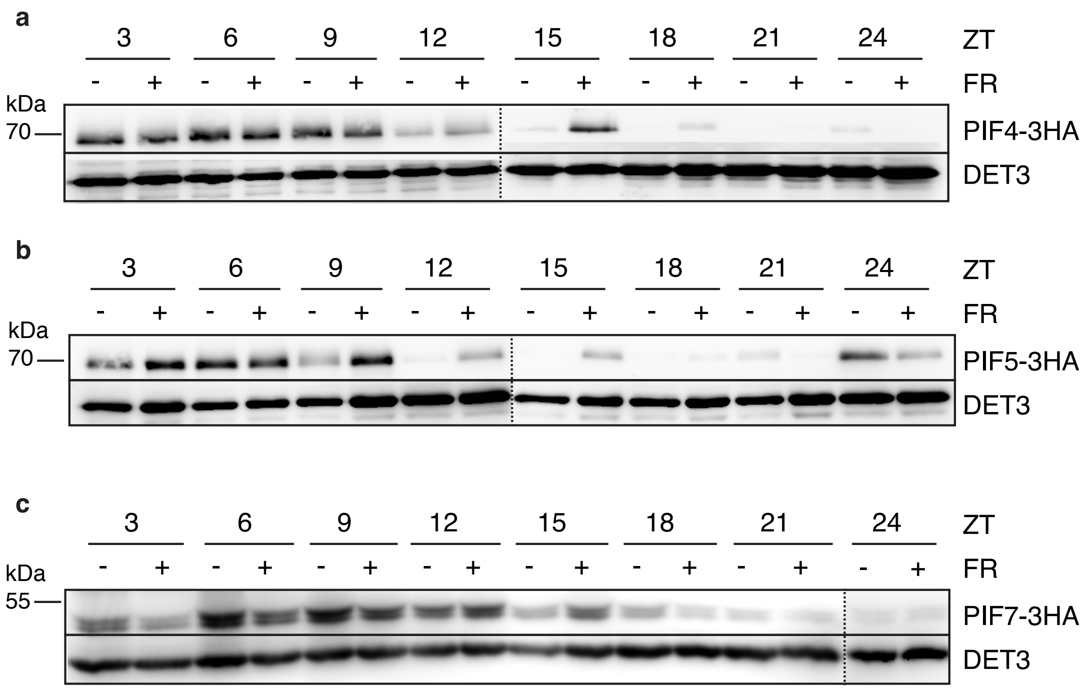


**Supplementary Figure 13**

PIF4, PIF5 and PIF7 protein accumulation in high and low R/FR. Western-blot of *pif4-101/pPIF4::PIF4-3HA* **a**, *pif5-3/pPIF5::PIF5-3HA* **b** and *pif7-2/pPIF7::PIF7-3HA* **c**. Plants were grown on soil for 5 days in high R/FR and either kept in control high R/FR or shifted to low R/FR in day 6. Samples were harvested every 3 hours in high and low R/FR of 10-day-old plants for 24 hours. Western-blots were performed using anti-DET3 and anti-HA antibodies.

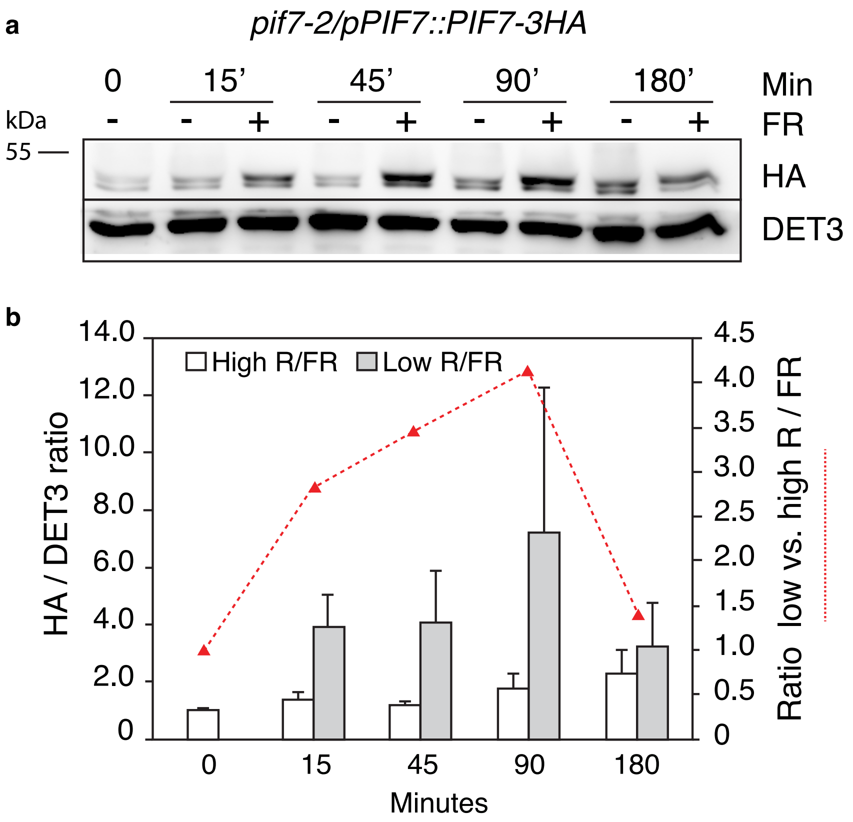


**Supplementary Figure 14**

Short-term PIF7-HA protein accumulation by western-blot in 6-day-old *pif7-2/pPIF7::PIF7-3HA* plants in high and low R/FR **a**, and quantitative analysis over DET3 protein levels **b**. Plants were grown for 5 days in high R/FR and in day 6 either kept in control high R/FR or shifted to low R/FR at ZT2. Samples were harvested before (0) and 15, 45, 90 and 180 minutes after shift to low R/FR. White (high R/FR) and grey (low R/FR) bars correspond to the average protein levels of 3 biological replicates and at least 3 technical replicates relative to DET3. Red dashed line represent the PIF protein level ratio of low/high R/FR. Error bars represent standard deviation. FR, supplemental far-red light. Note that we observe 2 isoforms for PIF7 with more of the slower migrating one present after transfer to low R/FR.

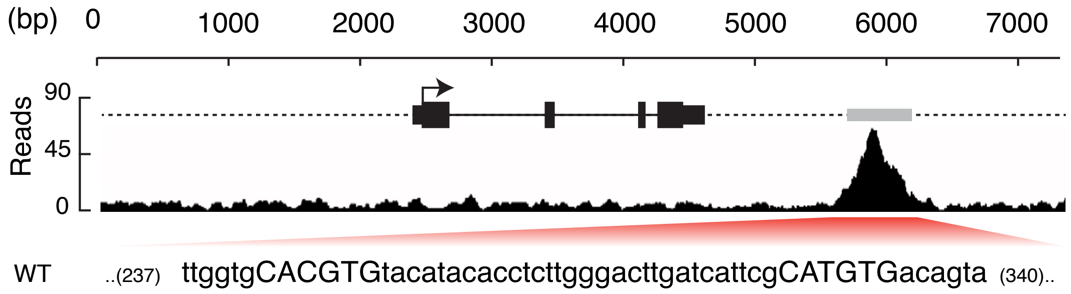


**Supplementary Figure 15**
Representation of PIF4 ChIP-seq reads mapped to the *FT* locus (At1g65480) [3]. Grey box represents high confidence PIF4 binding peak at the *FT* terminator and nucleotide sequence represent the WT DNA sequence. G-box (CACGTG) and PBE-box (CATGTG) are represented in uppercase.

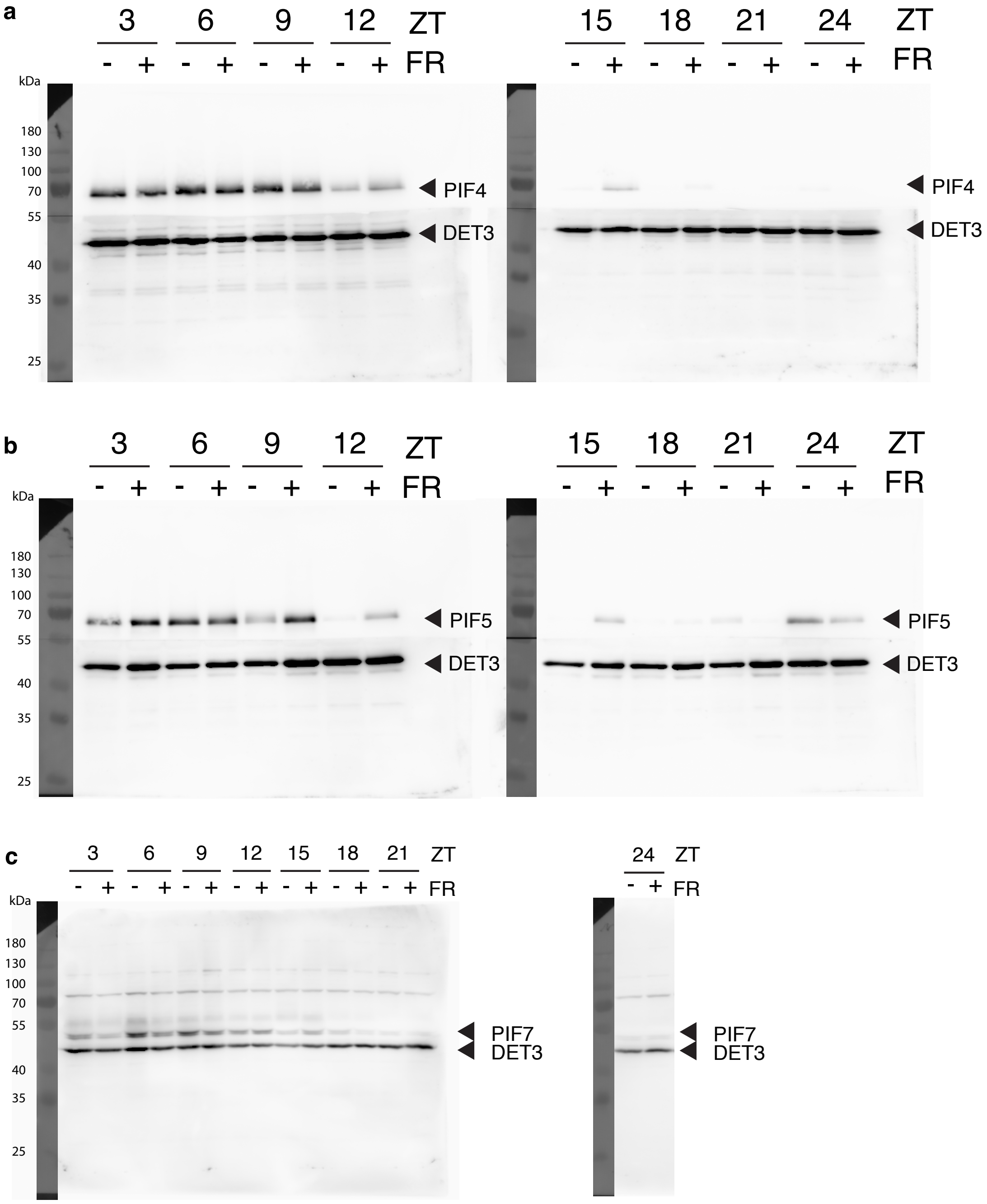


**Supplementary Figure 16**

Full western-blots shown in supplementary Figure 13a-c. **a.** PIF4-HA and DET3 loading control. **b.** PIF5-HA and DET3 loading control. **c.** PIF7-HA and DET3 loading control.

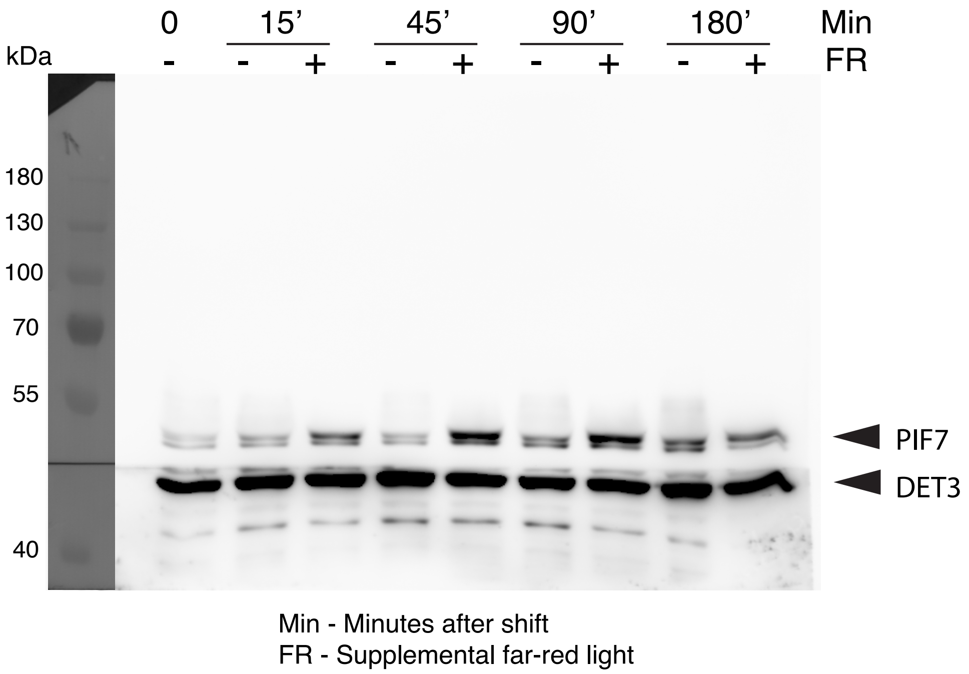


**Supplementary Figure 17**
Full western-blot shown in supplementary Figure 14a.

## Supplementary Table 1 – List of oligonucleotides used in this study.

| Oligos used for cloning | | | |
|---|---|---|---|
| **Construct** | **Backbone/Description** | **Oligonucleotide** | **Sequence (5'>3')** |
| pASF-02 | *pBGW/pPIF7::PIF7-3HA:tPIF7* | oASF-25 | AGCATGCGACGTCGGGCCCTAATCAAAATTACTTATATCAATGAAACTATATTTG |
| | | oVCG-165 | CTGGAACGTCGTATGGGTAGTCATCTCTTTTCTCATGATTCGAAG |
| | | oVCG-166 | CTTCGAATCATGAGAAAAGAGATGACTACCCATACGACGTTCCAG |
| | | oVCG-167 | CAATAATACTAGGTCGCTAGACTATCCTGCATAGTCCGGGAC |
| | | oVCG-168 | GTCCCGGACTATGCAGGATAGtctagcgacctagtattattg |
| | | oASF-26 | CGAGGGTACCCGGGGATCCTGTGCATATGATCTATTAACATTGAAG |
| pVG-24 | *pMAL-c2 TEV/MBP-PIF7_bHLH* | oVCG-226 (SacI) | CGAGCTCGAATGGACGACGGGGACGAGCAG |
| | | oVCG-192 (XbaI) | CCTCTAGACTTCATTGTTGGTGTTGGATGCTG |
| pVG-55 | *pGREENII-0800/pTSF::fLUC* | oVCG-440 (XhoI) | CCCCCCTCGAGGTCGACGGGTTTTTTTCTCGGTCATAAACG |
| | | oVCG-441 (NotI) | GGTGGCGGCCGCTCTAATTTATCTTGGATCTCAAGTATCTC |
| pVG-84 | *pGREENII-0800/pTSF 1xmut::fLUC* | .. | .. |
| | | .. | .. |
| pVG-87 | *pGREENII-0800/pTSF 3xmut::fLUC* | oVCG-374 | CAATGCCCGGATTTATCTCTCTGT |
| | | oVCG-559 | GACCAGTTTCATTTGCTGACCAC |
| | | oVCG-378 | GTGGTCAGCAAATGAAACTG |
| | | oASF-73 | TTCTTATTCCAAAACCCACCAG |
| | | oVCG-560 | CTGGTGGGTTTTGGAATAAG |
| | | oVCG-381 | ATTTATCTTGGATCTCAAGTATCTC |
| | | oVCG-440 (XhoI) | CCCCCCTCGAGGTCGACGGGTTTTTTTCTCGGTCATAAACG |
| | | oVCG-441 (NotI) | GGTGGCGGCCGCTCTAATTTATCTTGGATCTCAAGTATCTC |
| pAM-01 | *pCB308/pPIF4::GUS* | SL-128 (BamHI) | ATCGGATCCGCCGCCAATCTGCCGACAAG |
| | | SL-129 (BamHI) | ATCGGATCCGCTAGCGTCAGATCTCTGGAGACAT |
| pAM-03 | *pCB308/pPIF5::GUS* | SL-132 (BamHI) | ATCGGATCCAAATACAACGACATCATTTG |
| | | SL-133 (BamHI) | ATCGGATCCGCTAGCGTCAGATCTGTAAAGACACT |
| pMK-09 | *pCB308/pPIF7::GUS* | MK-207 (XbaI) | CCGTCTAGAAAAGAGTCGAACAGGGAAAGTTC |
| | | MK-208 (BamHI) | CCGGGATCCGTGTTACTTAGGCCGCACG |

| Oligos used for genotyping | | | |
|---|---|---|---|
| **Allele** | **Collection** | **Oligonucleotide** | **Sequence** |
| ***phyB-9*** | Point mutation | PB9 | GTGTCTGCGTTCTCAAAACG |
| | | B9dCAPS | GTGGAAGAAGCTCGACCAGGCTTTG |
| ***ft-10*** | GK-290E08 | oVCG-53 | CCCATTTGGACGTGAATGTAGACAC |
| | | oVCG-417 | CGAGCAACATGTTTGAGCTAT |
| ***co-101*** | SAIL_24_H04 | oVCG-61 | TAGCATCTGAATTTCATAACCAATCTCGATACAC |
| | | oVCG-143 | AGCTCCCACACCATCAAACTTACTACATC |
| ***tsf-1*** | SALK_087522 | oVCG-55 | ACGTGGACTCTCGTAGCACAC |
| | | oVCG-56 | ATTTTGCCGATTTCGGAAC |
| ***pif3-1*** | SALK_030753 | CF-230 | AGGCATTCCCATACCCATTG |
| | | oVCG-56 | ATTTTGCCGATTTCGGAAC |
| ***pif4-101*** | SAIL_114_G06 | SL-43 | CAGACGGTTGATCATCTG |
| | | oVCG-61 | TAGCATCTGAATTTCATAACCAATCTCGATACAC |
| ***pif5-3 (pil6-1)*** | SALK_087012 | SL-46 | TCGCTCACTCGCTTACTTAC |
| | | oVCG-56 | ATTTTGCCGATTTCGGAAC |

| | | | |
|---|---|---|---|
| ***pif7-1*** | CS68809 | SL-195 | GTGGCAAGTTGGCTCTTAGG |
| | | SL-169 | TGATAGTGACCTTAGGCGACTTTTGAACGC |
| ***pif7-2*** | SAIL_622_G02 | oASF-27 | GGAGAGCCATAGAGTTGG |
| | | oVCG-61 | TAGCATCTGAATTTCATAACCAATCTCGATACAC |

| **Oligos used for RT-qPCR** | | | |
|---|---|---|---|
| **Target** | **Primer Efficiency** | **Oligonucleotide** | **Sequence** |
| *FT* | 2.00 | oVCG-27 | CCCTGCTACAACTGGAACAAC |
| | | oVCG-28 | CACCCTGGTGCATACACTG |
| *TSF* | 2.00 | MT-608 | TGGAGATGTTCTTGATCCTTTC |
| | | MT-609 | TAGATCCAAGCCATTAGTAACC |
| *CO* | 2.05 | MT-596 | AACGCCTGCACCGTGTATTG |
| | | MT-597 | ACGCGATTGGCAGAGTGAAC |
| *PIF4* | 2.02 | oVCG-246 | TACCTCGATTTCCGGTTATGGATC |
| | | oVCG-247 | GTTGTTGACTTTGCTGTCCCGC |
| *PIF5* | 2.01 | oVCG-588 | GAGCAGCTCGCTAGGTACATG |
| | | oVCG-589 | GTTGTTGTTGCACGGTCTG |
| *PIF7* | 1.99 | SL-194 | AAAGGAGACGGCGTGATAGG |
| | | SL-195 | GTGGCAAGTTGGCTCTTAGG |
| *PIL1* | 1.92 | MT-125 | TCAGACTCAGGCTACTTCTTTTACTCA |
| | | MT-126 | TCCTCTATATTGCATTGCATCTTCTAA |
| *IAA29* | 1.94 | MT-157 | CTTCCAAGGGAAAGAGGGTGA |
| | | MT-158 | TTCCGCAAAGATCTTCCATGTAAC |
| *YUC8* | 2.00 | MT-271 | GGCGGCTTGTCTCCATGAAC |
| | | MT-171 | ACTGTTGACCTCGACGTTGTTG |
| UBC | 1.94 | UBC-F | CAGTCTGTGTGTAGAGCTATCATAGCAT |
| | | UBC-R | AGAAGATTCCCTGAGTCGCAGTT |
| YSL8 | 2.00 | YSL8-F | TCATTCGTTTCGGCCATGA |
| | | YSL8-R | CTCAGCAACAGACGCAAGCA |
| PIF7 - Pair 1 | 1.99 | SL-194 | AAAGGAGACGGCGTGATAGG |
| | | SL-195 | GTGGCAAGTTGGCTCTTAGG |
| PIF7 - Pair 2 | 1.97 | oASF-52 | GAACCACCCAAAGAAGCGTA |
| | | oASF-53 | CTGATCCATACAGCGCCTTT |
| PIF7 - Pair 3 | 1.94 | oASF-63 | TAGGTCGCTAGACTAATCTC |
| | | oASF-53 | CTGATCCATACAGCGCCTTT |

## Supplementary Table 2 – DNA sequences.

| **pTSF_3xmut synthesis** |
|---|
| tttttgtggtcagcaaatgaaactggtctgcaatagtttttagaagtctttatcggtttatttct**catgGg**ttttgtcagaaatttcttagaa atttttgtcttccaaacttggggaactaaatttgtttggagtgtaagaatacacggttgagcttttaggtaatacacttgtttttgaaaggt ataatgaattttaaaaaaattactttatcaacatttaagatttggagacaattttgaaatcacgtttgaaaacgaatttttttggccaattgtt cacttttctgtcgttattatacttctgttaccttgtgtctatttgttaatttacttgttttatttgatttactccattgttttaaaagttttactggttt gttttgttaaattgatatttttgatggtattaattaagtttagtactacattaagaaaagaaaaaaaaagagtctagtacgtatgtacacac caaccagatatgtgaacaattatggcgtcaatttatctggagcatatatacttttatatgatggtttaggttgaaggagttattctaaaat gcattaacatcatacataatcggtgtaatgtagtgtccttaactatttaaatgagtga**catgGg**acgcaaaatgcgtgacgtacgca aatatgcagcatattatatgcttgtgacattattttaaatagttcatgaagttacaatgataagtcagaataaattacatactagtggcta gcaagaaa**caagGg**gatgatgtataatttggaatatgattatttctgttttatactggtgggttttggaataagaatactataaatttag aaaaaaggaaagaaaaaaaaaacatg |

| **PIF7_bHLH** |
|---|

aatggacgacggggacgagcagcagcgattcacaacgagtccgaaaggagacggcgtgataggataaaccagaggatgag
aacacttcagaagctgcttcctactgcaagtaaggcggataaagtctcaatcttggatgatgttatcgaacacttgaaacagctaca
agcacaagtacagttcatgagcctaagagccaacttgccacaacaaatgatgattccgcaactacctccaccacagtcagttctca
gcatccaacaccaacaa

## References


1. Krzywinski M, Altman N. Visualizing samples with box plots. *Nat. Methods* **11**, 119-120 (2014).

2. Kohnen MV*, et al.* Neighbor detection induces organ-specific transcriptomes, revealing patterns underlying hypocotyl-specific growth. *Plant Cell* **28**, 2889-2904 (2016).

3. Pedmale UV*, et al.* Cryptochromes interact directly with PIFs to control plant growth in limiting blue light. *Cell* **164**, 233-245 (2016).
