# Supplementary material for: PIF transcription factors link a neighbor threat cue to accelerated reproduction in Arabidopsis
Source: Nat Commun. 2019 Sep 5;10:4005. doi: 10.1038/s41467-019-11882-7 (PMC6728355; doi:10.1038/s41467-019-11882-7)
Supplement: Supplementary file 3 — Reporting Summary [file 41467_2019_11882_MOESM3_ESM.pdf]

nature research

Corresponding author(s): Christian Fankhauser

Last updated by author(s): Jul 18, 2019

Reporting Summary

Nature Research wishes to improve the reproducibility of the work that we publish. This form provides structure for consistency and transparency in reporting. For further information on Nature Research policies, see Authors & Referees and the Editorial Policy Checklist .

Statistics

For all statistical analyses, confirm that the following items are present in the figure legend, table legend, main text, or Methods section.

n/a	Confirmed	
☐	☒	The exact sample size (*n*) for each experimental group/condition, given as a discrete number and unit of measurement
☐	☒	A statement on whether measurements were taken from distinct samples or whether the same sample was measured repeatedly
☐	☒	The statistical test(s) used AND whether they are one- or two-sided<br>*Only common tests should be described solely by name; describe more complex techniques in the Methods section.*
☒	☐	A description of all covariates tested
☒	☐	A description of any assumptions or corrections, such as tests of normality and adjustment for multiple comparisons
☒	☐	A full description of the statistical parameters including central tendency (e.g. means) or other basic estimates (e.g. regression coefficient) AND variation (e.g. standard deviation) or associated estimates of uncertainty (e.g. confidence intervals)
☒	☐	For null hypothesis testing, the test statistic (e.g. *F*, *t*, *r*) with confidence intervals, effect sizes, degrees of freedom and *P* value noted<br>*Give P values as exact values whenever suitable.*
☒	☐	For Bayesian analysis, information on the choice of priors and Markov chain Monte Carlo settings
☒	☐	For hierarchical and complex designs, identification of the appropriate level for tests and full reporting of outcomes
☒	☐	Estimates of effect sizes (e.g. Cohen's *d*, Pearson's *r*), indicating how they were calculated

Our web collection on statistics for biologists contains articles on many of the points above.

Software and code

Policy information about availability of computer code

Data collection	ImageQuant LAS 4000 mini (GE healthcare) was used for blot imaging; GloMax 96 Microplate Luminometer (Promega) for dual-luciferase assay; QuantStudio 6 Flex Real-Time PCR System (Applied Biosystems) for qPCR experiments; Ocean Optics USB2000+ spectrometer for light measurements; Thermochron iButtons (Maxim Integrated Products) for temperature monitoring; DNA microarray Scanner (Agilent Technologies) for protein-binding microarray; Leica M205 FCA Binocular.
Data analysis	Biogazelle qBase software for RT-qPCR analysis; TrimGalore (v0.3.7), bwa (v 0.7.15), picard tools (v 2.9.0), Samtools (v 1.3), bam2wig, wigToBigWig (Bio-BigFile-1.01), Genome Viewer for public ChIP-seq data analysis; Image Studio Lite (Li-Cor biosciences, v 2.5.2) for western-blot quantifications; Feature Extraction software (v 9.0, Agilent Technologies); PBM Analysis Suite (Berger & Bulyk, 2009 - Nature Protocols); Scripts were modified to adapt them to different custom microarray dimensions and Feature Extraction input files (Franco-Zorilla et al., 2014 - PNAS).

For manuscripts utilizing custom algorithms or software that are central to the research but not yet described in published literature, software must be made available to editors/reviewers. We strongly encourage code deposition in a community repository (e.g. GitHub). See the Nature Research guidelines for submitting code & software for further information.

Data

Policy information about availability of data

All manuscripts must include a data availability statement. This statement should provide the following information, where applicable:

- Accession codes, unique identifiers, or web links for publicly available datasets
- A list of figures that have associated raw data
- A description of any restrictions on data availability

All genetic resources generated in this work will be made available for the community upon direct request.

Field-specific reporting

Please select the one below that is the best fit for your research. If you are not sure, read the appropriate sections before making your selection.

- [x] Life sciences
- [ ] Behavioural & social sciences
- [ ] Ecological, evolutionary & environmental sciences

For a reference copy of the document with all sections, see nature.com/documents/nr-reporting-summary-flat.pdf

Life sciences study design

All studies must disclose on these points even when the disclosure is negative.

Sample size	For flowering phenotype analysis, sample sizes are described on each figure. RT-qPCR were performed using at least 3 independent biological replicates, each constituted of at least 15 pooled plants. Dual luciferase transient expression in N. benthamiana were performed using four biological replicates, each corresponding to two leaf discs from independent infiltrations . Western-blots were performed in biological triplicates, each using 15 seedlings.
Data exclusions	For TSF 24 hours diel RT-qPCR analysis CT values above the experimental controls were excluded. No data points were excluded from other experiments.
Replication	All experiments were replicated.
Randomization	The different genotypes were randomized for flowering phenotype, RT-qPCR, western-bot and GUS staining. For Dual luciferase transient expression, samples were randomly harvested from the infiltrations using different plants.
Blinding	Unknown number identities for the different genotypes were used for flowering phenotyping experiments.

Reporting for specific materials, systems and methods

We require information from authors about some types of materials, experimental systems and methods used in many studies. Here, indicate whether each material, system or method listed is relevant to your study. If you are not sure if a list item applies to your research, read the appropriate section before selecting a response.

Materials & experimental systems

n/a	Involved in the study	
[ ]	[x]	Antibodies
[x]	[ ]	Eukaryotic cell lines
[x]	[ ]	Palaeontology
[x]	[ ]	Animals and other organisms
[x]	[ ]	Human research participants
[x]	[ ]	Clinical data

Methods

n/a	Involved in the study	
[x]	[ ]	ChIP-seq
[x]	[ ]	Flow cytometry
[x]	[ ]	MRI-based neuroimaging

Antibodies

Antibodies used	Monoclonal anti-HA-Peroxidase (Roche, Cat. 12013819001)<br>Policlonal anti-DET3 antibody was provided by Karin Schumacher and it has been described in Schumacher et al., 1999 - Genes & Development 13 (24): 3259-3270.<br>Anti-rabbit IgG HRP-conjugated secondary antibody (Promega, Cat. W4011)
Validation	The anti-HA and anti-rabbit IgG HRP were validated by the respective manufacturers and anti-DET3 has been validated in Schumacher et al., 1999 - Genes & Development 13 (24): 3259-3270.
